# Cuproptosis signature and PLCD3 predicts immune infiltration and drug responses in osteosarcoma

**DOI:** 10.3389/fonc.2023.1156455

**Published:** 2023-03-16

**Authors:** Hai Hu, Yuesong Yin, Binbin Jiang, Zhennan Feng, Ting Cai, Song Wu

**Affiliations:** ^1^ Department of Orthopedics, The Third Xiangya Hospital, Central South University, Changsha, China; ^2^ Department of Gastroenterology, The Third Xiangya Hospital, Central South University, Changsha, China

**Keywords:** osteosarcoma, cuproptosis, immune infiltration, tumor microenvironment, drug response, machine learning

## Abstract

Osteosarcoma (OS) is a cancer that is frequently found in children and adolescents and has made little improvement in terms of prognosis in recent years. A recently discovered type of programmed cell death called cuproptosis is mediated by copper ions and the tricarboxylic acid (TCA) cycle. The expression patterns, roles, and prognostic and predictive capabilities of the cuproptosis regulating genes were investigated in this work. TARGET and GEO provided transcriptional profiling of OS. To find different cuproptosis gene expression patterns, consensus clustering was used. To identify hub genes linked to cuproptosis, differential expression (DE) and weighted gene co-expression network analysis (WGCNA) were used. Cox regression and Random Survival Forest were used to build an evaluation model for prognosis. For various clusters/subgroups, GSVA, mRNAsi, and other immune infiltration experiments were carried out. The drug-responsive study was carried out by the Oncopredict algorithm. Cuproptosis genes displayed two unique patterns of expression, and high expression of FDX1 was associated with a poor outcome in OS patients. The TCA cycle and other tumor-promoting pathways were validated by the functional study, and activation of the cuproptosis genes may also be connected with immunosuppressive state. The robust survival prediction ability of a five-gene prognostic model was verified. This rating method also took stemness and immunosuppressive characteristics into account. Additionally, it can be associated with a higher sensitivity to medications that block PI3K/AKT/mTOR signaling as well as numerous chemoresistances. U2OS cell migration and proliferation may be encouraged by PLCD3. The relevance of PLCD3 in immunotherapy prediction was verified. The prognostic significance, expressing patterns, and functions of cuproptosis in OS were revealed in this work on a preliminary basis. The cuproptosis-related scoring model worked well for predicting prognosis and chemoresistance.

## 1 Introduction

Osteosarcoma (OS) continues to be the most prevalent primary bone cancer in children and adolescents, although being uncommon globally (1). With 4.4 instances per million people in the US, this tumor reaches its peak incidence in adolescence, which is consistent with a pubertal growth surge (2). Patients with OS have a >60% five-year survival rate thanks to the present conventional therapeutic approach of surgery and chemotherapy, but since 1980, little has been learned about the pathophysiology and targeted therapy of OS. Patients with metastatic disease and relapse cannot benefit from additional surgery or chemoradiotherapy (3). In-depth research on novel etiology and treatment targets for OS is urgently needed given the non-negligible severe socioeconomic burden on young people.

The tailored treatment of OS may greatly benefit from further study into programmed cell death (PCD), which is still a hot topic in oncology. For instance, cisplatin, a traditional first-line chemotherapeutic treatment for OS, induces apoptosis (4). By inducing oxidative stress dependent on GSH depletion and ROS overproduction, ferroptosis promoters such as phenethyl isothiocyanate (PEITC), baldachin, and ursolic acid have been identified as potential adjuvant chemotherapy treatments (5–7). Similar to this, inhibiting RIP1- and RIP3-dependent necroptosis effectively reduced lung metastasis and tumor growth in an OS mouse model (8).

A new PCD variant called cuproptosis was published in March 2022 by Peter T et al. (9). The buildup of monovalent copper ions may interact directly with proteins that have been lipoylated, which are mostly found in the mitochondria that power the TCA cycle. The loss of proteins containing the Fe-S cluster and the production of acute proteotoxic stress as a result of copper chelating lipoylated protein aggregation led to an independent type of cell death. For oncology researchers interested in copper toxicity in the treatment of cancer, this result is encouraging. A significant anti-tumor effect in patients with low plasma lactate dehydrogenase (LDH) was revealed in the phase 3 clinical trial to apply copper ionophores for melanoma, suggesting malignancies with a high dependence on mitochondrial metabolism were likely to benefit from cuproptosis-related molecular therapies (10).

The metabolic reprogramming in OS (11) is characterized by abnormally suppressed TCA cycle and high levels of oxidized glutathione (GSH), and GSH regulates copper ion cytotoxicity by inhibiting the oxidation of divalent copper ions to monovalent copper ions (9). It is important to talk about the activities of the lipoylation and cuproptosis pathways. In this investigation, we seek to identify functional pathways and genetic targets closely associated with cuproptosis, investigate the expression patterns of cuproptosis regulatory genes, and assess the influence of these targets on the prognosis of OS patients. Additionally, immune infiltrates landscapes, chemotherapeutic responsiveness, and cancer stem-like cellular features are also implicated in identifying their distinctions in patients with various cuproptosis patterns. This study might offer initial recommendations and a feasibility analysis for treatment plans that aim to treat copper toxicity in OS patients.

## 2 Materials and methods

### 2.1 Dataset obtaining and processing

In this investigation, public transcriptional profiling datasets from OS patients were used, including the TARGET OS dataset and the GSE21257 dataset from GEO. For the TARGET OS dataset, the GDC portal (https://portal.gdc.cancer.gov/), along with complete clinical information and RNA expression data in raw count and TPM format, were downloaded by GDC client. Expression data in TPM format was then converted into a log2(TPM+1) matrix for further analysis, and 85 samples with full RNA expression and clinical data were finally included. The URL for GSE21257 was https://www.ncbi.nlm.nih.gov/geo/. 53 samples with complete information were eventually included after starting with raw data and moving on to obtain a normalized expression matrix and clinical data using the R package beadarray and illuminaHumanv2.db. R (version 4.1.3) and Bioconductor programs for data cleaning and gene analysis were used to analyze all the aforementioned data signature annotation.

### 2.2 Cuproptosis regulatory gene set and unsupervised consensus clustering

The cuproptosis regulatory gene set was obtained from the latest literature by Peter T et al. (9), including *FDX1*, *LIAS*, *LIPT1*, *DLD*, *DLAT*, *PDHA1*, *PDHB*, *MTF1*, *GLS* and *CDKN2A*. The sweep() function in R was used to normalize the expression matrix for these genes in log2(TPM+1) format before package ConsensusClusterPlus was used for unsupervised clustering. The study’s parameters were maxK = 4, reps = 500, pItem = 0,8, pFeature = 1, title = title, clusterAlg = hc, and distance = canberra. Each clustering was evaluated using the consensus CDF value and CDF curve delta area.

### 2.3 Differential expressing analysis

The TARGET OS dataset’s expression data in raw count format and the R package DEseq2 were used for the DE analysis. Briefly, grouping information was first established using results from previous clustering; next, the entire expression matrix in TARGET OS was pre-screened to remove genes with zero expression in more than 20% of samples; finally, a DEseqDataSet object was built; the DESeq() function was used to calculate DE fold change and perform a significance test. FDR 0.05 was the cutoff for identifying genes as significantly differentially expressed (DE), and these genes were referred to as cuproptosis-related DE genes (CRDEGs).

### 2.4 Weight gene correlation network analysis and identification of cuproptosis-related hub genes

To find additional genes connected to cuproptosis clustering, WGCNA was carried out using DE genes. Hierarchical clustering analysis was first performed using the hclust tool. Then, the pickSoftThreshold duty during module construction screened the soft thresholding power setting (6 in this study). Various modules’ average connectivity degrees and independence were tested using candidate power (1 to 30). A suitable power value was chosen if the autonomy level was greater than 0.8. Co-expression networks (modules) were built using the WGCNA R package (The R package WGCNA is a collection of functions for calculating various weighted association analyses, which can be used for network construction, gene screening, gene cluster identification, topological feature calculation, data simulation, and visualization). The minimum module size was set to 30, giving each module a distinct color label. On the basis of its correlation with clusters, the core module was chosen. Genes in the core module with GS values greater than 0.8 and Module Membership (MM)>0.5 was defined as hub genes, termed cuproptosis-related hub genes (CRHGs).

### 2.5 Construction and validation of the cuproptosis-related prognostic 00predicting model

Based on the aforementioned CRHGs, a Random Survival Forest (RSF) plus Cox regression algorithmic technique was used for the selection of predictive features, model development, and internal and external validation. Details are as follows:

#### 2.5.1 Univariate Cox regression for preliminary feature screening

TARGET_OS dataset was first randomly divided into the train (70%) and internal test (30%) datasets by createDataPartition() function in the R package caret. Univariate Cox regression analysis was then applied for all CRHGs by R package survival and survminer. Given the low sample volume for the TARGET_OS dataset, a bootstrap (12) sampling strategy was adopted: in 1,000 replicates of sampling with replacement, a gene was proved as prognosis-related only when the univariate cox regression results showed FDR< 0.05 for more than 900 times; this step was accomplished by sample() function in R.

#### 2.5.2 RSF model for prognostic genes selection

The randomforestSRC (13) R package’s rfsrc() function was used to access the remaining genes in order to build an RSF model. The optimal values were ntree=1000, block.size=1, mtry=2, nodesize=13, splitrule=“logrank” after adjustments. The var.select() function was used to choose features based on minimal depth in order to build the final prognostic model. As cuproptosis-related prognostic genes, these genes (CRPGs).

#### 2.5.3 Prognostic model construction by multivariate Cox regression

A multivariate Cox regression model was built based on CRPGs. Coefficients in this regression were applied for a final cuproptosis-related prognostic scoring (CRP score) model calculated as follows:


$$CRP\;score=\sum_{i=1}^{n}{\text{Coef}}_{i}*{\text{x}}_{i}$$

Where Coefi was the coefficient of multivariate Cox regression and xi was the log2(TPM+1) expression value corresponding to the No.i CRPG.

#### 2.5.4 Model validation

Then, for patients in TARGET OS for train and internal tests and GSE21257 for external validation, the CRP score was determined. The timeROC package represented the Time-dependent ROC curve, and the area under the curve (AUC) was used as the foundation for evaluating the CRP score model’s ability to predict outcomes.

### 2.6 Gene set variation analysis

GSVA was used by the R package GSVA and GSEAbase to investigate various enrichment statuses in gene function for distinct clusters and subgroups. Two gene sets, c2.cp.kegg.v7.4.symbols and h.all.v7.4.symbols, were used for functional annotation from MsigDB (http://www.gsea-msigdb.org/gsea/msigdb/). After that, the LIMMA package was used to identify the enrichment variations between various subgroups (13–15).

### 2.7 Calculation of the stemness index (mRNAsi)

Based on the mean-centered gene expression profiles of PSCs in the PCBC database (syn2701943), the stemness signature was derived *via* the one-class logistic regression (OCLR) machine learning algorithm (16), which was also verified by leave-one-out cross-validation. Then, we calculated the Spearman correlations between the normalized expression matrix of OS samples and the stemness signature. Eventually, the stemness index was identified by scaling the Spearman correlation coefficients to be between 0 and 1. The higher the mRNAsi, the greater the tumor dedifferentiation and higher stemness (17).

### 2.8 Compound resistance and sensitivity analysis

Genomics of Drug Sensitivity in Cancer (GDSC, http://www.cancerrxgene.org/downloads) database (18), which contained drug sensitivity data (IC50) of 1,000 cell lines, was accessed to get drug sensitivity and resistance information for osteosarcoma cell lines. Then R package Oncopredict (19) based on the Ridge Regression algorithm was applied to predict the drug response of samples in the TARGET_OS cohort. Spearman correlation analysis was performed to calculate the correlation between drug sensitivity and CRP_Score. The absolute value of correlation coefficient > 0.4 and FDR< 0.05 were regarded as significant.

### 2.9 Analysis for immune cell infiltration and immune signatures

Following the usual analysis procedure, we first used the ESTIMATE program in R to evaluate the stromal purity and general immune infiltration of tumor samples. For the investigation of tumor immune cell infiltration, we used the algorithms CIBERSORT and ssGSEA (13, 20, 21). The original publications’ archives with the defining gene signatures for each type of immune cell were obtained.

### 2.10 q-PCR experiment

The primers used for q-PCR are as follows: β-actin (https://www.ncbi.nlm.nih.gov/gene/60; F ACCCTGAAGTACCCCATCGAG; R AGCACAGCCTGGATAGCAAC). PLCD3 (https://www.ncbi.nlm.nih.gov/gene/113026; F CTCATTCGGGAGGCAGGGAA; R CTGGGGACTGTAGTTGGCTG). The cell groups are as follows: NC, si-PLCD3-1, si-PLCD3-2, and si-PLCD3-3.

### 2.11 Transwell experiment

In DMEM with 10% FBS and 1% double antibody, U2OS cells were grown. Pancreatic enzymes were used to digest the U2OS cells at the logarithmic growth stage before being counted and distributed uniformly in six-well plates with roughly 1x105 cells per well. The cells were transfected with NC and si-PLCD3 on the second day. The cells were switched to a full medium for 48 hours after 6 hours, and they were then cultured in an EDU37°C incubator overnight. Paraformaldehyde was used to fix the samples after collection. To defrost on ice, remove the necessary si-PLCD3 and NC. They took four sterile tubes. A total of 95 uL of serum-free MEM/DMEM media was added to two tubes. The tubes were then filled with 5 uL of NC and 5 uL of Lip2000, respectively. The equivalent centrifuge tubes received the addition of si-PLCD3 in the same manner. Mix gently, then set aside for five minutes at room temperature. Following a 20-minute rest period at room temperature, combine the two tubes. Finally, the mixture was blended and added uniformly to the transfection hole. Replace with fresh and full culture medium six hours after starting the culture in incubators at 37°C. The following are the cell groups: Si-PLCD3-1 and Si-PLCD3-2, NC. Corning sold the Transwell chamber (3428), which was acquired.

### 2.12 EdU experiment

In DMEM with 10% FBS and 1% double antibody, U2OS cells were grown. Pancreatic enzymes were used to digest the U2OS cells at the logarithmic growth stage before being counted and distributed uniformly in six-well plates with roughly 1x105 cells per well. The cells were transfected with NC and si-PLCD3 on the second day. The cells were switched to a full medium for 48 hours after 6 hours, and they were then cultured in an EDU37°C incubator overnight. Paraformaldehyde was used to fix the samples after collection. To defrost on ice, remove the necessary si-PLCD3 and NC. They took four sterile tubes. A total of 95 uL of serum-free MEM/DMEM media was added to two tubes. The tubes were then filled with 5 uL of NC and 5 uL of Lip2000, respectively. The equivalent centrifuge tubes received the addition of si-PLCD3 in the same manner. Mix gently, then set aside for five minutes at room temperature. Following a 20-minute rest period at room temperature, combine the two tubes. Finally, the mixture was blended and added uniformly to the transfection hole. Replace with fresh and full culture medium six hours after starting the culture in incubators at 37°C. The following are the cell groups: Si-PLCD3-1 and Si-PLCD3-2, NC. Ribo supplied the EdU kit (RN: R11078.2).

### 2.13 Statistical analysis

All statistical calculations were done in R. (version 4.1.3). The comparison of count data was assessed using Fisher’s test and the Chi-square test. The Student-t test was used for measurement data with a normal distribution, whereas the Wilcox test was used for data with an abnormal distribution. All correlation investigations must be completed using Spearman analysis. The Kaplan-Meier survival curve was represented using the R package survival and survminer.

## 3 Results

The workflow chart of the study is shown in **Supplement Figure 1**.

### 3.1 Distinct expression patterns for cuproptosis regulatory genes were identified in osteosarcoma patients

**Figure 1:**
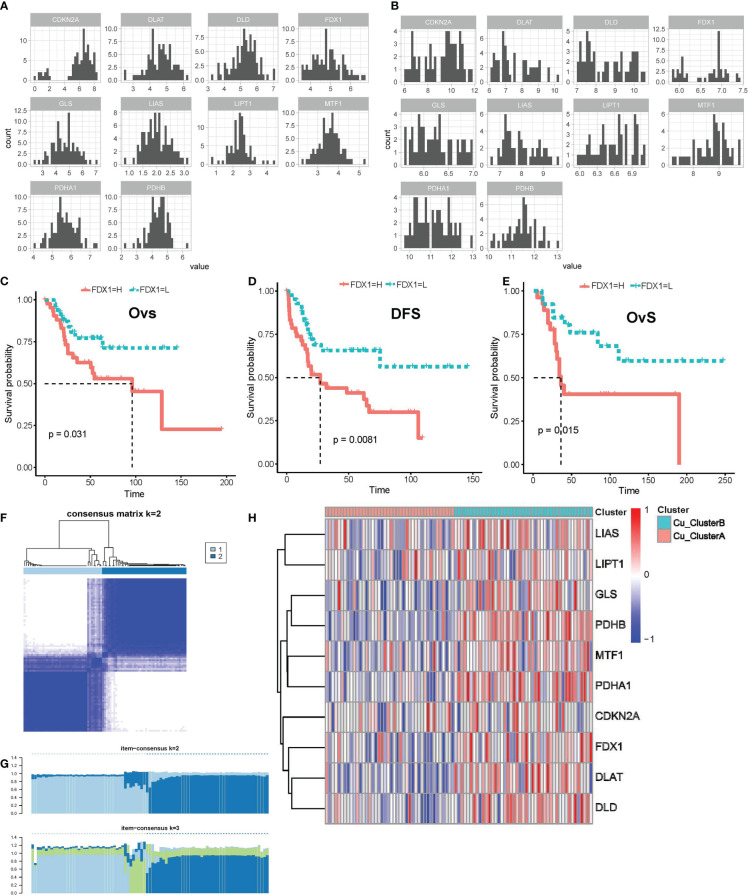
Cuproptosis regulatory genes were expressed in distinct patterns in OS samples. **(A, B)** Expression distributions of cuproptosis regulatory genes in TARGET_OS **(A)** and GSE21257 **(B)** datasets. **(C–E)** K-M survival curve for FDX1 high- and low- expression subgroups in TARGET_OS **(C, D)** and GSE21257 datasets **(E)**, Ovs, overall survival; DFS, disease-free survival. **(F, G)** Results of consensus clustering based on the expression of cuproptosis regulatory genes, **(F)** Consensus heatmap, **(G)** Item-Consensus plot. **(H)** Heatmap shows the expression of cuproptosis genes in distinct patterns.

We initially examined the expression pattern of the genes that regulate cuprotosis based on the log2(TMP+1) expression matrix. All 10 genes were expressed in the TARGET OS and GSE21257 datasets, as seen in **Figures 1A, B**, and their expression followed a normal distribution, which gave us the foundation for further investigation. By combining the Kaplan-Meier survival curve and univariate Cox regression with survival data, we found that elevated FDX1 expression is linked to both lower overall survival (OvS) and disease-free survival (DFS) in OS patients (**Figures 1C–E**). These findings suggested that cuproptosis might contribute to the malignant biological activity of OS since FDX1 was shown to be a key regulator in cuproptosis by linking the cytotoxicity of cooper ions and protein lipoylation in the TCA cycle.

Following that, unsupervised consensus clustering was carried out using the expression matrix of 10 genes involved in cuproptosis. Eighty-five samples in TARGET OS were best grouped into two clusters, referred to as Cu ClusterA (42 samples) and Cu ClusterB (43 samples), as shown in **Figures 1F, G**. The samples in Cu ClusterB tended to overexpress all cuproptosis genes, as seen in **Figure 1H**, whereas CDKN2A appeared to be indistinguishable. These results showed that the cuproptosis pathway genes’ activity in OS patients showed two different patterns.

### 3.2 Cuproptosis clusters in OS patients represented differences in immune infiltration and stemness properties

**Figure 2:**
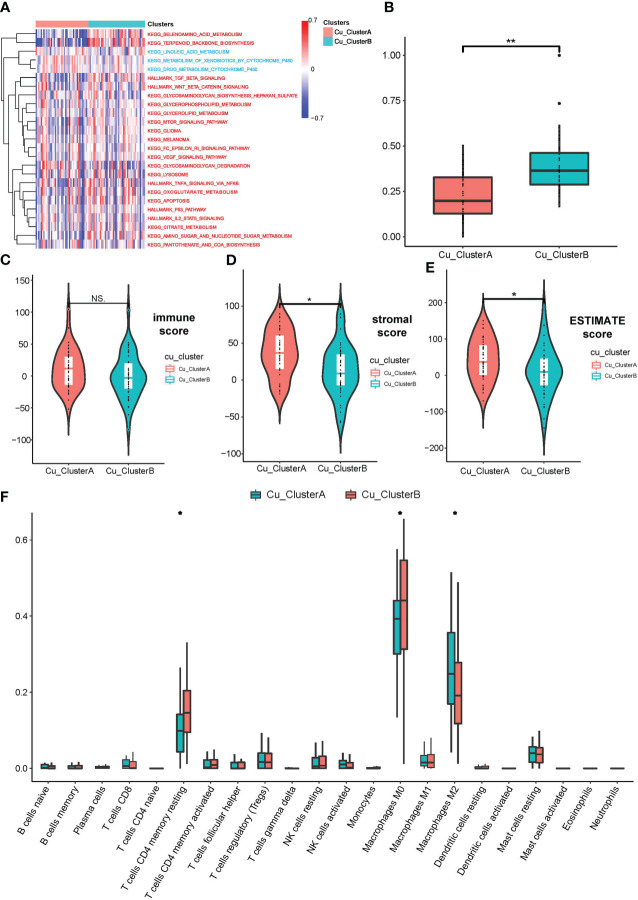
OS samples in different Cu_Clusters exhibited distinct tumor biological characteristics. **(A)** GSVA analysis showed diverse enriched pathways in different Cu_Clusters. **(B)** Divergence in the mRNAsi index showed differences in stemness properties between Cu_ClusterA and Cu_ClusteB. **(C–E)** ESTIMATE analysis for the overall status of immune cell infiltration and stromal component samples in the TARGET_OS dataset. **(F)** ssGSEA for the infiltration analysis of 29 types of immune cells in different Cu_Clusters *P < 0.05; **P < 0.01.

Then, we pondered how the two clusters’ malignant biological characteristics varied from one another. So, we carried out a number of functional investigations. The expression of cuproptosis genes was typically active in Cu ClusterB samples, according to GSVA analysis, which first revealed a number of pathways that were sparked. TCA cycle-related pathways (such as citrate metabolism, oxoglutarate metabolism, and pantothenic acid biosynthesis) and traditional cancer-promoting pathways (such as TGF-, WNT/-catenin, p53, and IL./STAT5 signalings) are two categories of important findings (**Figure 2A**). We developed the mRNAsi index to show the difference in cellular stemness between the two clusters because the majority of these enriched pathways were involved in the destiny control of cancer stem-like cells (CSLCs). Cu ClusterB displayed a substantially higher mRNAsi than Cu ClusterA, as illustrated in **Figure 2B**, indicating that enhanced cuproptosis gene expression may function as an initiating factor in immortal proliferation, quick metastasis, and chemo-resistance linked to CSLC activities.

In addition, we carried out a number of researches on immunological infiltration between two clusters. **Figures 2C–E** illustrates how the ESTIMATE approach revealed that samples in Cu ClusterB had lower stromal scores than Cu ClusterA, indicating that Cu ClusterB had fewer stromal components. Samples in Cu ClusterA tended to enhance activated immune cells, according to CIBERSORT and ssGSEA for a study of just one type of immune cell (e.g., Activated CD4 T cell, dendritic cell, and Macrophages M2). In contrast, models in Cu ClusterB (such as Regulatory T cell, MDSC, and Macrophages M0) may show signs of a dormant immunological state (**Figure 2F** and **Supplement Figure 2A**).

### 3.3 Screening of cuproptosis-related genes revealed a functional connection between cuproptosis and other biological processes in OS

**Figure 3:**
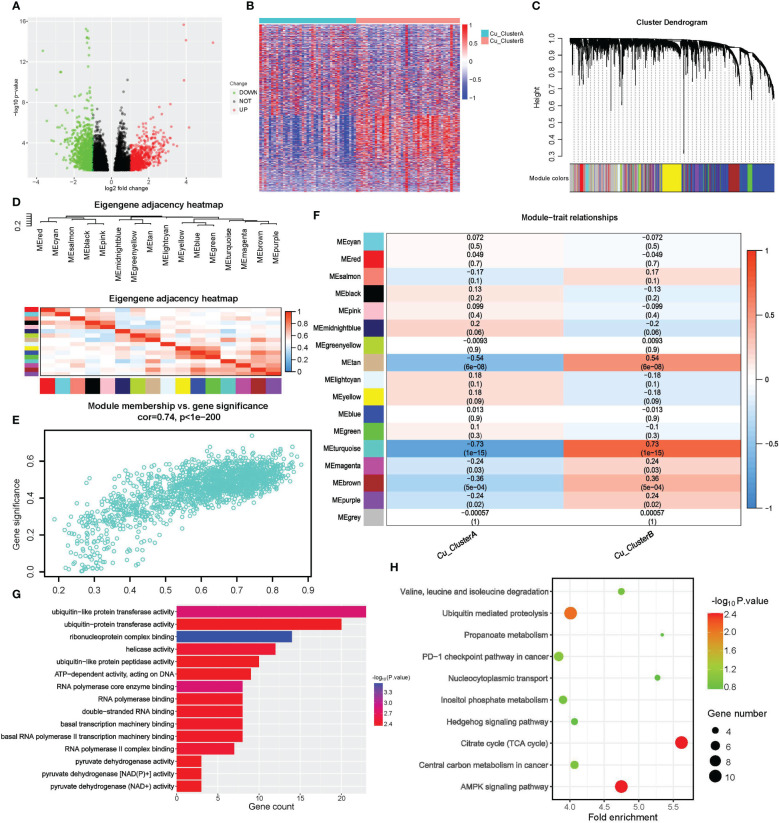
Screening of cuproptosis-related genes by DE analysis and WGCNA. **(A, B)** Results of DE analysis in different Cu_Cluseters; **(A)** Volcano plot of DEGs; **(B)** Heatmap of DEGs. **(C–F)** WGCNA analysis for DEGs to identify gene module that was most correlated with Cu_Clusters; **(G, H)** Pathway enrichment analysis for hub genes obtained from WGCNA; **(G)** Molecular function analysis of WGCNA hub genes in GO **(H)** Pathway enrichment analysis of WGCNA hub genes in KEGG.

We first performed a differential expression (DE) study to find CRGs associated with cuproptosis clusters. A total of 6537 genes, including 3565 up-regulated genes in Cu ClusterA and 2972 up-regulated genes in Cu ClusterB, matched the criteria for DE, as shown in **Figures 3A, B**. WGCNA analysis was used, using DEGs as input objects, to further narrow down the potential genes highly connected with cuproptosis clusters, and 16 modules were ultimately discovered (**Figures 3C, D**). Notably, the cuproptosis clusters had the strongest correlation with Module turquoise (MEturquoise), which had 1762 genes and had a R = 0.73 with Cu ClusterB, p = 1e-15, in **Figure 3E**. Further verification showed that genes in MEturquoise had strong consistency in principal component representation (shown by Module Membership, MM) and external connection with cuproptosis clusters (indicated by Module Membership, MM) (**Figure 3F**).

We eventually discovered 331 hub genes in MEturquoise based on the selection criteria of GS>0.8 and MM>0.5 described above. For further examination, these signatures were classified as CRGs. For CRGs, enrichment analysis was used to investigate the co-regulated pathways and biological processes. Notably, as shown in **Figures 3G, H**, the KEGG analysis revealed a high enrichment of the TCA cycle and NAD(P)+ activity pathways, further demonstrating the close relationship between cuproptosis and the TCA cycle. The terms RNA synthesis, metabolism & splicing, and ubiquitin-proteasome pathway also commonly appeared in search results. Traditional methods of controlling cuproptosis are suggested by AMPK and Hedgehog signaling. The emergence of the PD-1 checkpoint pathway suggested that cuproptosis might contribute to the responsiveness of tumor treatment.

### 3.4 Selection of cuproptosis-related prognostic genes and construction of cuproptosis-related prognostic score model

**Figure 4:**
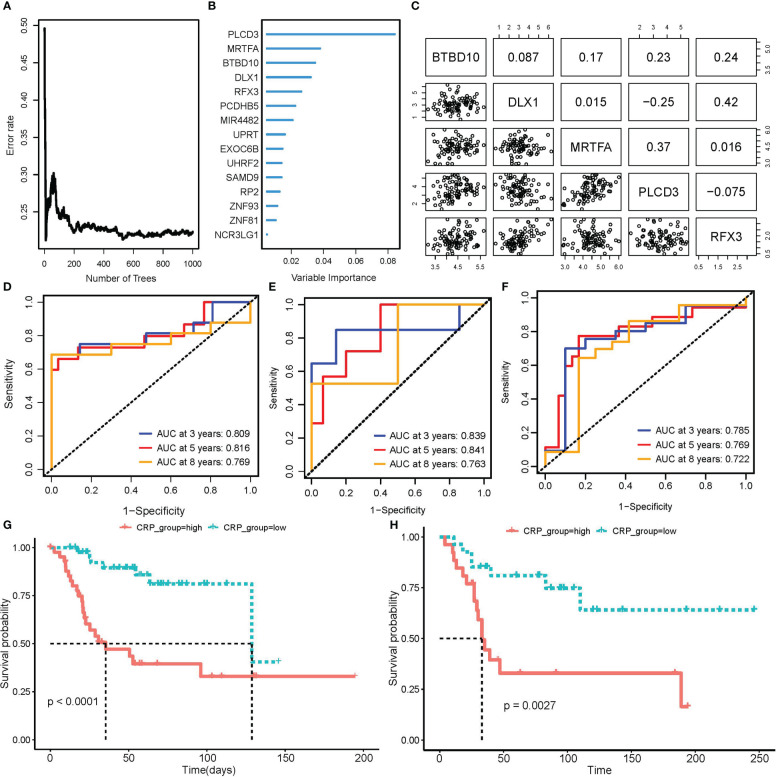
Construction and validation of CRP score model. **(A, B)** RSF model training and variable selection; **(A)** error rate trends as the number of trees increased when training RSF model; **(B)** Variable importance of selected features. **(C)** Correlation of expression in 5 genes that RSF selected to train CRP score model. **(D–F)** Time-dependent ROC curve to test the predictive ability of CRP score for OS patients in TARGET_OS train set **(D)**, test set **(E)**, as well as GSE21573 external validation set **(F)**. **(G, H)** K-M curve of CRP scores high and low subgroups for patients’ overall survival in TARGET_OS **(G)** and GSE21573 **(H)** datasets.


Patients in the TARGET OS cohort were 7:3 randomly split between the training and testing groups. 16 prognostic CRGs were left after performing univariate Cox regression with bootstrap sampling to reduce redundancy based on CRGs. Then, using a 1,000-tree random survival forest model, the minimum depth values selected the five gene signatures that would ultimately be used as CRPGs: BTBD10, DLX1, MRTFA, PLCD3, and RFX3 (**Figures 4A, B**). The scatter plot revealed no obvious association between the expression of these five genes, ruling out model redundancy in the process (**Figure 4C**). The CRP score model was then created by performing multivariate Cox regression using CRPGs:

$$\begin{array}{l} CRP\;score=(-1.8626130)*Exp_{BTBD10}+0.2978399*Exp_{DLX1}-0.9252084*Exp_{MRTFA}\\ +0.1514946*Exp_{PLCD3}+1.0547832*Exp_{RFX3} \end{array}$$

For each patient in a train, test, and external validation dataset, we computed a CRP score. In these datasets, we used a time-dependent ROC curve to find the prediction power for overall survival. The area under the curve (AUC) was convincingly confirmed in the TARGET testing set GSE21257 validation set and reached 0.809 at three years, 0.816 at five years, and 0.769 at eight years (**Figures 4D–F**). Based on the median score, OS patients were divided into CRP score high and CRP score low subgroups. The K-M curve further demonstrated that OS patients with higher CRP scores had considerably worse OvS times (**Figures 4G, H**).

### 3.5 Correlation analysis between CRP score and malignant biological behaviors

**Figure 5:**
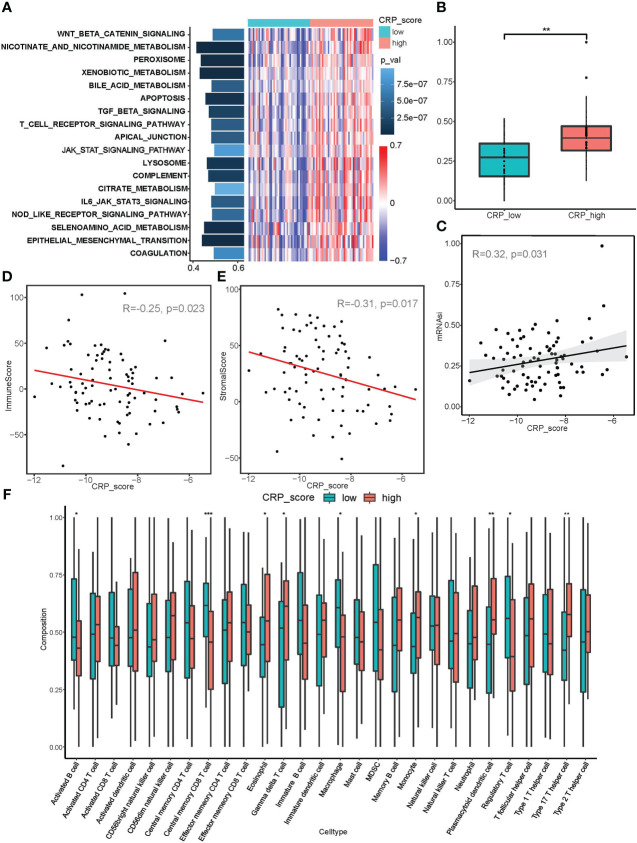
Correlation analysis between CRP score and malignant biological behaviors. **(A)** GSVA analysis showed diverse enriched pathways between CRP_high and CRP_low subgroups. **(B, C)** mRNAsi index analysis revealed differences in stemness properties between CRP_high and CRP_low subgroups and a significant correlation between CRP_score and mRNAsi. **(D, E)** ESTIMATE analysis for the correlation between CRP_score and immune cell infiltration as well as a stromal component in samples of the TARGET_OS dataset. **(F)** ssGSEA for the infiltration analysis of 29 types of immune cells in CRP_high and CRP_low subgroups *P < 0.05; **P < 0.01; ***P < 0.001.

We also carried out a number of functional studies. First, GSVA analysis indicated that the CRP high subgroup was enriched for various cancer-promoting pathways, including Wnt/-catenin, TGF-, and JAK/STAT signaling, which overlapped with Cu ClusterB. Improvements were made to the TCA cycle-related pathways, demonstrating the coherence between Cu Clusters and CRP subgroups. Notably, the CRP high fraction also had activation of the epithelial-mesenchymal transition (EMT) pathway, suggesting that samples with poorer prognoses were more likely to develop distant metastases (**Figure 5A**).

Additionally, the mRNAsi index was used in connection studies with CRP results. In contrast to the CRP low subgroup, samples in the CRP high subgroup showed a considerably higher mRNAsi index (**Figure 5B**). Additionally, the TARGET OS dataset revealed a strong association between CRP score and mRNAsi in every person (R=0.32, p=0.031, **Figure 5C**), suggesting that OS samples with higher CRP values may have more pronounced stemness features.

According to the immune infiltration analysis, a higher CRP score was linked to immunosuppression (R = - 0.25, p = 0.023; **Figure 5D**); while a lower CRP score was linked to a greater stromal score (R = - 0.31, p = 0.017; **Figure 5E**). The CRP high subgroup was related with higher infiltration of Macrophages M0, Type 17 T helper cells, and T cells, according to an examination of infiltration for various immune cells. The CRP low subgroup, on the other hand, was connected to enhanced infiltration of Macrophages M2, Regulatory T cell, Central memory CD8 T cell, and Activated B cell, showing different immune infiltration patterns in OS patients (**Figure 5F** and **Supplement Figure 2B**). It should be highlighted that the CRP low subgroup showed increased expression of PDL1, TIM3, and TIGIT (**Supplement Figure 2C**). Because immunosuppression and CRP score are correlated, anti-PD-1/PD-L1 immunotherapeutic medicines may be more effective for OS patients with lower CRP values.

### 3.6 Correlation analysis between CRP score and malignant biological behaviors

From CCLE, 10 OS cell lines and their expression matrix were taken. In order to determine the link between the CRP score and the IC50 for each molecule contained in the GDSC v2 database, we first calculated the CRP score for these cell lines. The elevated CRP score was linked to greater resistance to a number of medications, particularly those that target the ERK/MAPK pathway and cell cycle, as shown in **Supplement Figure 3A**. Unexpectedly, cell lines with higher CRP ratings appeared to be more responsive to AT13148, a medication that blocks PI3K/Akt/mTOR signaling. To further forecast the pharmacological reactions of samples in the TARGET OS dataset, we utilized a machine learning system. Two medications that target the PI3K/mTOR pathway, AZD6482 and AZD8055, were probably more sensitive in OS samples with higher CRP scores, as demonstrated in **Supplement Figure 3B** and **Supplement Figure 2D**. Additionally, samples with high CRP values responded more favorably to linsitinib targeting IGF1R.

In light of the aforementioned findings, treating patients with high CRP scores who were thought to have bad prognoses may involve targeting PI3K/Akt/mTOR signaling. Contrarily, drugs that target the cell cycle and Wnt signaling pathways are frequently ineffective against patients with high CRP values. Given that most first-line chemotherapeutics for OS used cell cycle inhibition as their primary mechanism of action, the CRP score model may also be able to predict clinical chemoresistance in OS patients.

### 3.7 Pan-cancer analysis on model genes

**Figure 6:**
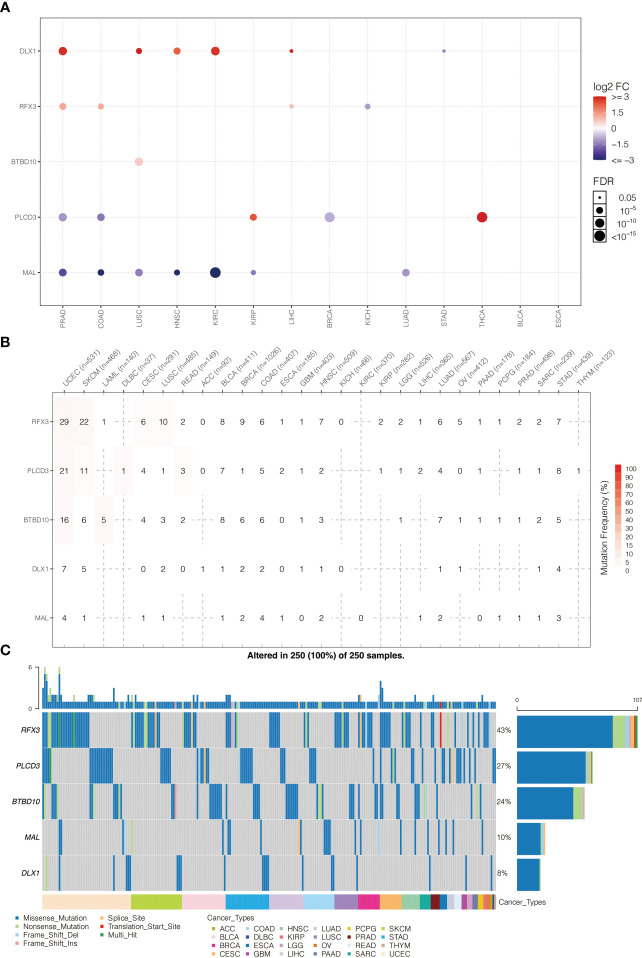
**(A)** Pan-cancer expression pattern of model genes. **(B)** Pan-cancer SNP analysis on model genes. **(C)** Pan-cancer SNP landscape on model genes.

The expression pattern of model genes in pan-cancer is shown in **Figure 6A**. BTBD10, DLX1, MRTFA, PLCD3, and RFX3 were highly expressed in PRAD, COAD, LUSC, HNSC, and KIRC. The somatic mutation frequency of model genes is shown in **Figure 6B**. BTBD10, DLX1, MRTFA, PLCD3, and RFX3 had relatively high mutation rates in UCEC and SKCM. The somatic mutation landscape of model genes is shown in **Figure 6C**. BTBD10 (43%), PLCD3 (27%), and RFX3 (24%) were frequently mutated in pan-cancer.

**Figure 7:**
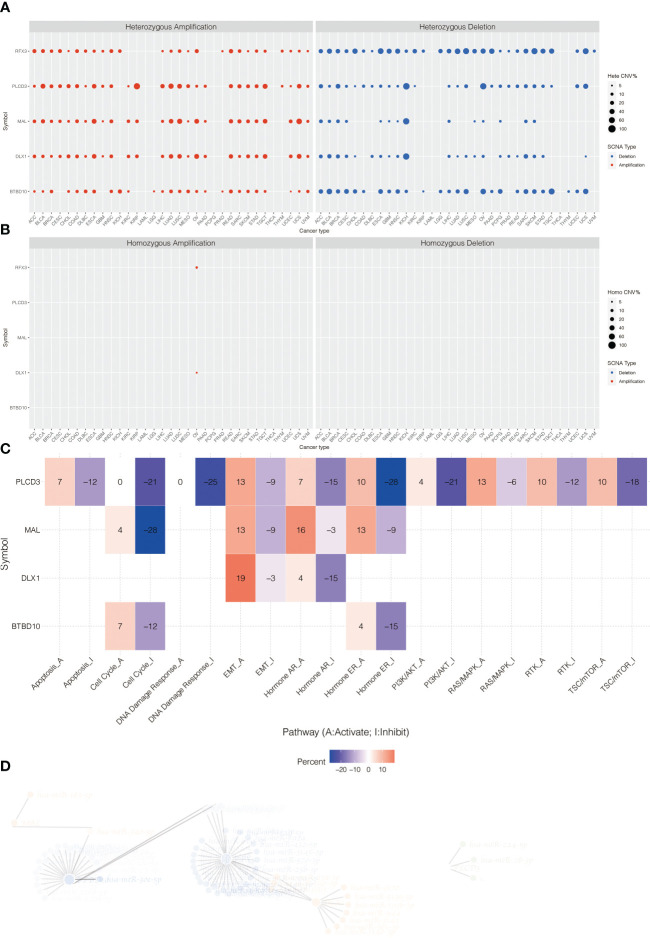
**(A)** The heterozygous CNV profiles (amplification and depletion) of model genes. **(B)** The homozygous CNV profiles (amplification and depletion) of model genes. **(C)** Pathway analysis related to PLCD3. **(D)** The miRNA regulation network of model genes.

The heterozygous CNV profiles (amplification and depletion) of model genes are shown in **Figure 7A**. The homozygous CNV profiles (amplification and depletion) of model genes are shown in **Figure 7B**. Pathway analysis revealed that PLCD3 was related to activated apoptosis, EMT, hormone AR, hormone ER, PI3K/Akt, RAS/MAPK, RTK, and TSC/mTOR (**Figure 7C**). The miRNA regulation network of model genes is shown in **Figure 7D**.

### 3.8 *In vitro* validation on PLCD3

**Figure 8:**
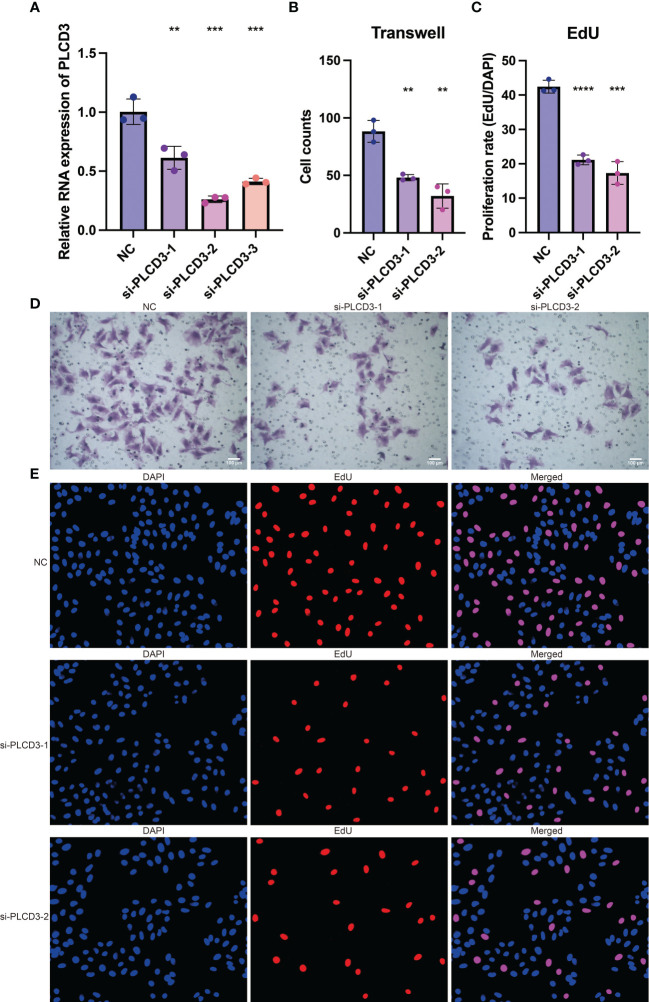
The tumor-promoting role of PLCD3. **(A)** The relative RNA expression of PLCD3 in NC and three si-RNA groups by q-PCR assay. **(B)** Statistical analysis of the cell counts in NC and two si-RNA groups by Transwell assay. **(C)** Statistical analysis of the proliferation rate (EdU/DAPI) in NC and two si-RNA groups by EdU assay. **(D)** The cell counts in NC and two si-RNA groups by Transwell assay. **(E)** The proliferation rate (EdU/DAPI) in NC and two si-RNA groups by EdU assay. **, P<0.01; ***, P<0.001; ****, P<0.0001.

The tumor-promoting activity of PLCD3 was investigated by *in vitro* tests since it is a crucial gene in the CRP score. Three si-RNA significantly reduced the relative RNA expression of PLCD3 in the NC and three si-RNA groups, according to a q-PCR experiment (**Figure 8A**). **Figure 8B** displays the statistical analysis of the cell counts in the NC and two si-RNA groups using the Transwell test. **Figure 8C** illustrates the statistical analysis of the proliferation rate (EdU/DAPI) in the NC and two si-RNA groups. Transwell assay representative photos of the cell counts in the NC and two si-RNA groups (**Figure 8D**), showing that the number of migrated cells was dramatically decreased in the two si-RNA groups. Typical pictures of the proliferation rate in NC (EdU/DAPI). Examples of the proliferation rate (EdU/DAPI) in the NC and two si-RNA groups by EdU test are shown in **Figure 8E**, where the positively stained cells in the two si-RNA groups were dramatically decreased.

### 3.9 Immunotherapy prediction of PLCD3

**Figure 9:**
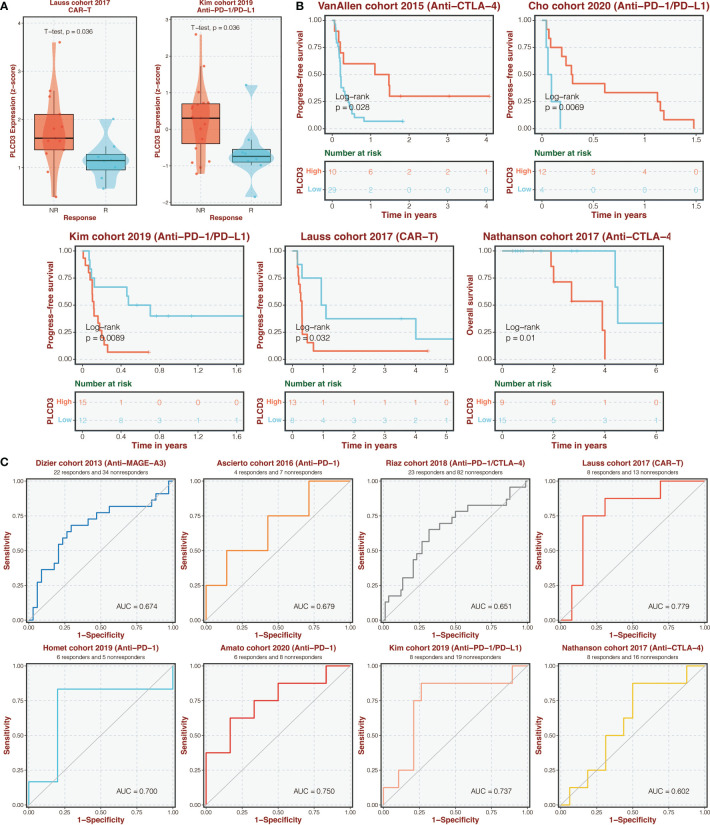
Immunotherapy prediction of PLCD3. **(A)** The expression of PLCD3 in responders and non-responders in immunotherapy cohorts. **(B)** Survival analysis was performed on the two groups regarding PLCD3 expression in immunotherapy cohorts. **(C)** The ROC curve of PLCD3 in predicting immunotherapy response in immunotherapy cohorts.

**Figure 9A** depicts the expression of PLCD3 in immunotherapy cohorts of responders and non-responders, with responders exhibiting higher expression of PLCD3 in the Lauss cohort of 2017 and Kim cohort of 2019. Regarding the two groups’ PLCD3 expression in immunotherapy cohorts, a survival analysis was carried out (**Figure 9B**). In the VanAllen cohort of 2015 and the Cho cohort of 2020, PLCD3 was linked to improved survival; in the Kim cohort of 2019, the Nathanson cohort of 2017, and the Lauss cohort of 2019, PLCD3 was linked to worse survival. In eight immunotherapy cohorts, PLCD3 demonstrated strong predictive power for immunotherapy response (**Figure 9C**).

**Figure 10:**
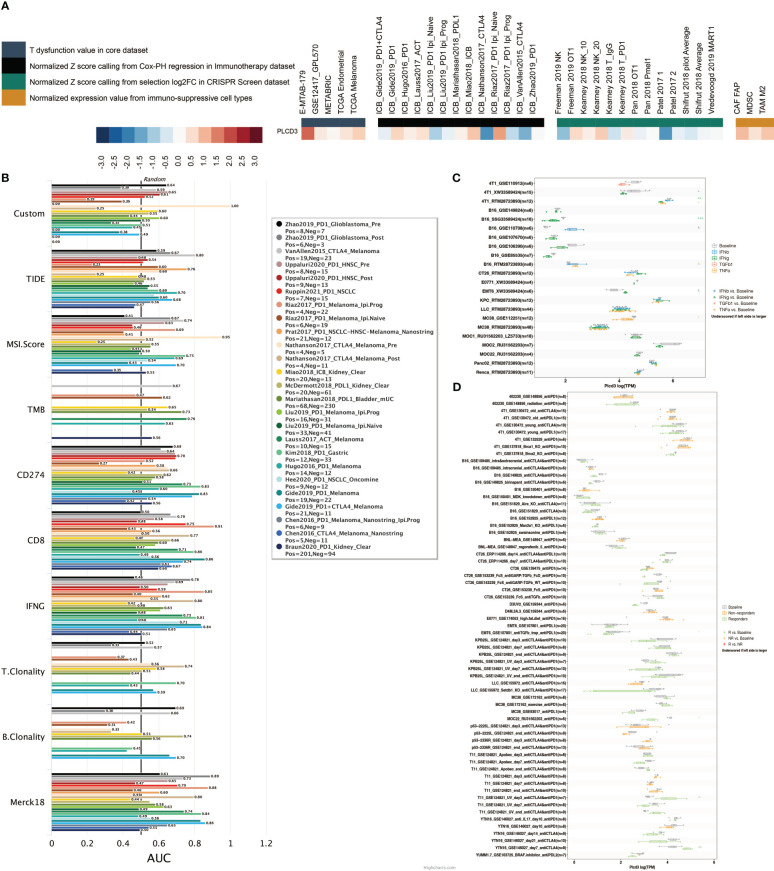
Immunotherapy prediction of PLCD3. **(A)** Regulator prioritization performed by TIDE. **(B)** Biomarker evaluation by TIDE. **(C)** Cytokine treatment prediction by TISMO. **(D)** Immunotherapy prediction by TISMO.

**Figure 10A** illustrates the relationship between PLCD3 and T dysfunction value (core dataset), normalized Z score calling from Cox-PH regression (immunotherapy datasets), normalized Z score calling from selection log2FC (CRISPR screening datasets), and normalized expression value from immune-suppressive cell types. PLCD3 had an AUC greater than 0.5 in ten immunotherapy cohorts with regard to its predictive value (**Figure 10B**). In seven mouse cohorts, the cytokine treatment prediction revealed that PLCD3 could strongly predict the treatment with cytokines (**Figure 10C**). In two mouse cohorts, the immunotherapy prediction demonstrated that PLCD3 could accurately predict immunotherapy (**Figure 10D**).

### 3.10 Protein interaction network, illness network, and pan-cancer immune infiltration pattern of PLCD3

**Figure 11:**
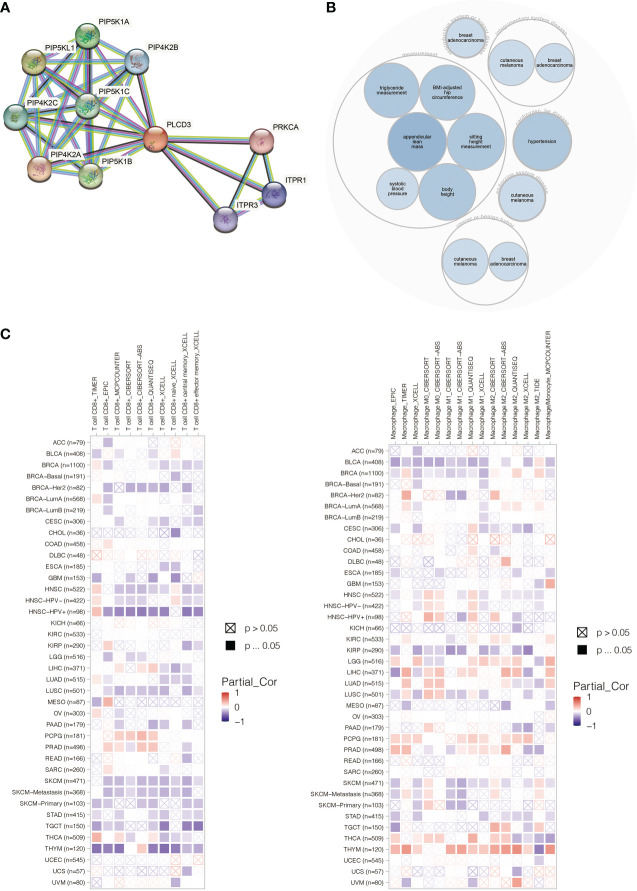
**(A)** The PLCD3 protein interaction network. **(B)** The PLCD3 illness network. **(C)** PLCD3’s pan-cancer immune infiltration pattern.

PLCD3 was found to interact with ITRP3, ITPR1, PRKCA, and PIP4K families by STRING (**Figure 11A**). PLCD3 was involved in hypertension, cutaneous melanoma, and breast adenocarcinoma by Open Targets Platform (**Figure 11B**). PLCD3 positively correlated with macrophages and negatively correlated with T cells in most cancers by TIMER (**Figure 11C**).

## 4 Discussion

Cuproptosis is a recently identified type of programmed cell death. Little is currently known about this unique, mitochondrial-dependent mechanism, however Peter T. et al. This work provides a preliminary description of the regulatory environment of cuproptosis-related pathways in osteosarcoma based on the available information. Some key findings may serve as an inspiration for work on OS and many other pathological conditions. First, it is found that OS patients have a poor prognosis and high FDX1 expression. FDX1 was first discovered as a mitochondrial electron transporter for cytochrome P450 metabolism (22, 23). Some sporadic studies identified FDX1 as a tumorigenesis regulator. In xenograft models of multi-tumors, Tsvepkov P et al. (24) proved that FDX1 worked as an oncogene rescuing elesclomol-induced cell death. Zhang Y et al. (25) found that FDX1 could regulate iron metabolism and mitochondrial homeostasis in tumor cells through the p53 pathway. Our work may inspire more research on FDX1 as a key element in cuproptosis and an oncogene to control the pathogenesis of OS because no studies on the association between FDX1 and OS have been located.

Our study has also thoroughly examined the regulatory pathways connected to cuproptosis and its potential roles in OS. Cuproptosis gene up-regulation resulted in the enrichment of a few well-known cancer-promoting pathways, including TGF-, Wnt/-catenin, and p53 signaling. These results might offer suggestions for further experiments on cuproptosis regulation pathways. Additionally, we discovered that cuproptosis may also generate an immunosuppressive state and CSLC characteristics. Ferroptosis and cuproptosis have certain molecular commonalities in several types of programmed cell death. Both were correlated with the reduction of metal ions and redox metabolic pathway mediated by GSH/NADPH in mitochondria (26, 27). Some recent studies have suggested that CSLCs might be sensitive to ferroptosis due to their relatively strong dependency on nutrition intake and higher intracellular levels of metal trace elements to maintain their self-renewal (28, 29). For tumor immunology, ferroptosis might also play a crucial role in regulating T cells. Ferroptosis induction in CD8+ and CD4+ T cells could lead to phospholipid hydroperoxide and impair its antitumor function (30, 31). These results are in line with our research, which show that cuproptosis-regulated gene activation is positively correlated with a higher mRNAsi index and an increase in the infiltration of immunosuppressive cells. Therefore, it is encouraging that future studies will concentrate on controlling cuproptosis in CSLCs and tumor microenvironments.

Our study identified five cuproptosis-related prognostic genes and built a reliable prognostic predicting model (CRP score model) based on them using a number of bioinformatic and machine learning methods. Our search revealed that studies on the role of these five genes, except MRTFA and RFX, in the etiology of OS had yet to be published. Matrix stiffness regulates EMT *via* cytoskeletal remodeling and MRTFA translocation in osteosarcoma (32). MRTFA is strongly associated with cell viability of its correlation with cytoskeleton and actin (33). It has been identified as an EMT and metastasis regulator in NPC (34) and NSCLC (35). BTBD10 functions as an activator of AKT family members by inhibiting PPP2CA-mediated dephosphorylation, and a few studies have identified it as a prognostic risk factor in hepatocellular carcinoma (36) and glioma (37). DLX1 serves as a two-sided transcriptional regulator of the TGF-β superfamily that may be either an oncogene or a suppressor in different types of tumors (38, 39). PLCD3 is a member of the phospholipase C family, which catalyzes the hydrolysis of phosphatidylinositol 4,5-bisphosphate to generate the second messenger diacylglycerol and inositol 1,4,5-trisphosphate (IP3) (40). PLCD3 is involved in the proliferation, migration, and invasion of nasopharyngeal carcinoma (41). PLCD3 inhibits apoptosis and promotes thyroid cancer’s proliferation, migration, and invasion *via* the Hippo pathway (42). As PLCD3 was not studied in osteosarcoma, the *in vitro* validation was performed on PLCD3. PLCD3 could facilitate the proliferation and migration of osteosarcoma. p53 could directly regulate target genes, including MDM2, TP53I3, and RRM2B, or indirectly regulate numerous further genes through several hub genes, including EHF and RFX, through various drug treatments in osteosarcoma (43). RFX3 is a transcription factor that is essential for the differentiation of nodal monocilia (44). It has been reported that these two genes may also be involved in malignant biological behaviors of cancers (42, 45), but the mechanisms are poorly understood. Given that drug responses were predicted for OS patients with varying CRP scores, this 5-gene prognostic model is not only deserving of exploration of their mechanism in cuproptosis regulation and OS tumorigenesis/progression but also potential for translational medical outcomes, particularly for the future targeted therapy targeting PI3K/AKT/mTOR signaling, as compounds targeting this pathway could remain highly sensitive in patients with high CRP scores (poor prognosis).

We had to acknowledge that this study has limitations as researchers in the fields of bioinformatics and machine learning. Since OS is a relatively uncommon tumor, it is challenging to gather WGS data, and the sample size is modest when compared to other cancer types. The TARGET database provided by GDC has to have certain types of data, including SNP, copy number variation, and protein expression profiling, completed or accessible. These flaws likely decreased the power of statistical tests throughout the study, particularly for machine learning-related studies like ridge regression and random survival forests. In a summary, our study demonstrated the distinctive cuproptosis regulatory gene expression profiles in osteosarcoma patients. It revealed some fresh information on the connections between this recently discovered kind of PCD and cancer-related pathways, stemness features, and immune infiltration traits. A scoring model based on cuproptosis-related clustering may have a significant impact on OS patient prognosis prediction and may influence clinical chemotherapy regimen selection and the creation of novel targeted medications.

## Data availability statement

The original contributions presented in the study are included in the article/**Supplementary Material**. Further inquiries can be directed to the corresponding authors.

## Author contributions

SW and TC designed the concept of this research. HH conducted all the studies in this paper. YY validated and proofread the data. HH wrote this paper. BJ and ZF revised this paper. All authors contributed to the article and approved the submitted version.

## Supplementary material

Figure S1 Workflow diagram of this study.

Figure S2 Figure S2 **(A)** The expression of immune cells in two Cu Clusters. **(B)** The expression of immune cells in two CRP score groups. **(C)** The expression of checkpoints in two CRP score groups. **(D)** The correlation of drug response and CRP score.

Figure S3 Figure S3 Pharmacogenomics Analysis of CRP score. **(A)** Signaling pathways targeted by drugs resistant or sensitive to CRP_score in OS cell lines; **(B)** Molecular targets and signaling pathways targeted by drugs resistant or susceptible to CRP_score in OS samples predicted by the Oncopredict algorithm.
