# Supplementary figures and images for: Cuproptosis signature and PLCD3 predicts immune infiltration and drug responses in osteosarcoma
Source: Front Oncol. 2023 Mar 16;13:1156455. doi: 10.3389/fonc.2023.1156455 (PMC10060837; doi:10.3389/fonc.2023.1156455)

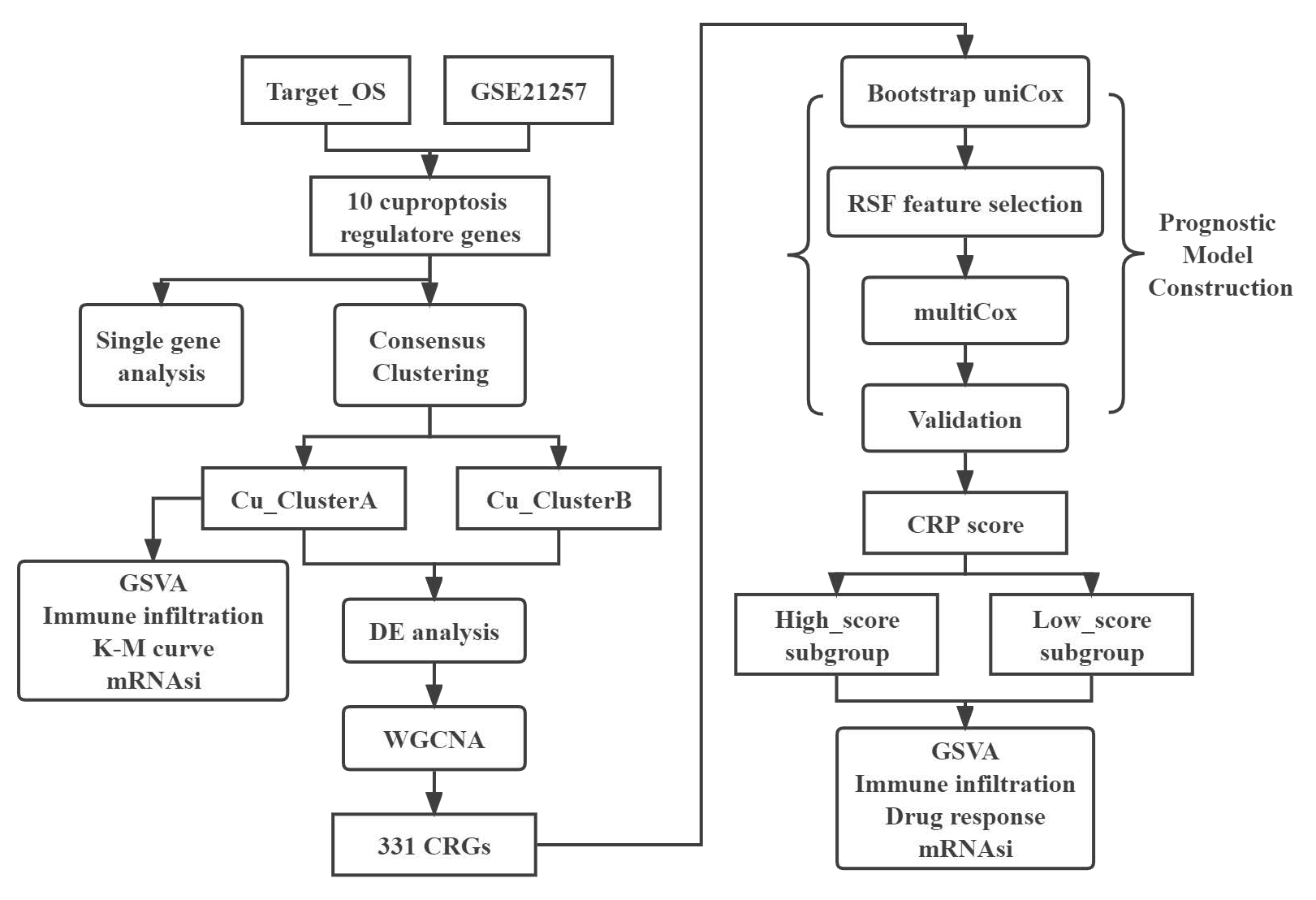

Supplement: Figure S1 — Workflow diagram of this study. [file Image_1.tif]

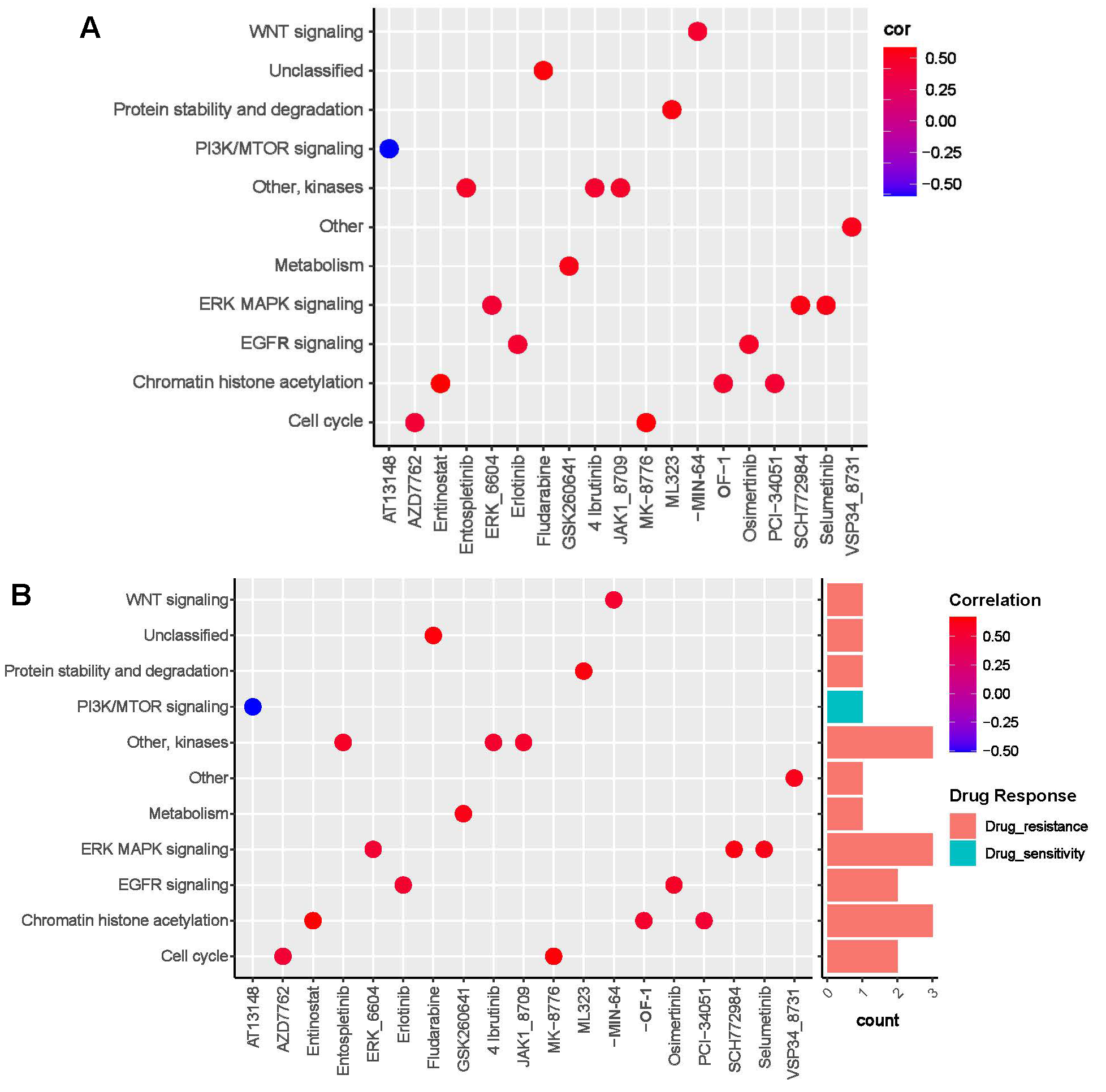

Supplement: Figure S3 — Pharmacogenomics Analysis of CRP score. (A) Signaling pathways targeted by drugs resistant or sensitive to CRP_score in OS cell lines; (B) Molecular targets and signaling pathways targeted by drugs resistant or susceptible to CRP_score in OS samples predicted by the Oncopredict algorithm. [file Image_3.tif]
